# Risk of central line-associated bloodstream infections during COVID-19 pandemic in intensive care patients in a tertiary care centre in Saudi Arabia

**DOI:** 10.1017/S0950268824000736

**Published:** 2024-06-03

**Authors:** Majid M. Alshamrani, Aiman El-Saed, Omar Aldayhani, Abdulaziz Alhassan, Abdullah Alhamoudi, Mohammed Alsultan, Mohammed Alrasheed, Fatmah Othman

**Affiliations:** 1 Infection Prevention and Control Department, King Abdulaziz Medical City, Riyadh, Saudi Arabia; 2 King Saud bin Abdulaziz University for Health Sciences, Riyadh, Saudi Arabia; 3 King Abdullah International Medical Research Center, Riyadh, Saudi Arabia; 4 Community Medicine Department, Faculty of Medicine, Mansoura University, Mansoura, Egypt; 5 College of Medicine, King Saud bin Abdulaziz University for Health Sciences, Riyadh, Saudi Arabia

**Keywords:** CLABSI, COVID-19, healthcare-associated infections, mortality, case fatality, Saudi Arabia

## Abstract

This retrospective study compared central line-associated bloodstream infection (CLABSI) rates per 1 000 central line days, and overall mortality before and during the COVID-19 pandemic in adult, paediatric, and neonatal ICU patients at King Abdul-Aziz Medical City-Riyadh who had a central line and were diagnosed with CLABSI according to the National Healthcare Safety Network standard definition. The study spanned between January 2018 and December 2019 (pre-pandemic), and January 2020 and December 2021 (pandemic). SARS-CoV-2 was confirmed by positive RT-PCR testing. The study included 156 CLABSI events and 46 406 central line days; 52 and 22 447 (respectively) in pre-pandemic, and 104 and 23 959 (respectively) during the pandemic. CLABSI rates increased by 2.02 per 1 000 central line days during the pandemic period (from 2.32 to 4.34, *p* < 0.001). Likewise, overall mortality rates increased by 0.86 per 1 000 patient days (from 0.93 to 1.79, *p* = 0.003). Both CLABSI rates (6.18 vs. 3.7, *p* = 0.006) and overall mortality (2.72 vs. 1.47, *p* = 0.014) were higher among COVID-19 patients compared to non-COVID-19 patients. The pandemic was associated with a substantial increase in CLABSI-associated morbidity and mortality.

## Introduction

Healthcare-associated infections (HAIs) are a significant concern in healthcare settings globally [1, 2]. While there are many guidelines for preventing HAIs, the impact of the COVID-19 pandemic on infection control practices has been challenging, placing a huge burden on healthcare systems to maintain surveillance activities considering the increases in critical care capacity and the surge of COVID-19 cases in intensive care units (ICU) with corresponding prolonged hospital stays [3].

A growing body of evidence has indicated that the pandemic led to an increase in the rates of several HAIs, particularly central line-associated bloodstream infections (CLABSI), catheter-associated urinary tract infections, ventilator-associated adverse events, and methicillin-resistant *Staphylococcus aureus* bacteraemia [4–6]. Several potential factors have been identified as contributors to this increased risk due to extended hospitalization periods, increased disease severity, and longer durations of indwelling device use [5].

According to the National Healthcare Safety Network (NHSN), monthly CLABSI rates increased on average from 0.40 before COVID-19 to 1.7 during the pandemic, while other reports indicated that the CLABSI rates increased by 71% (0.68 to 1.16 per 1 000-line days) in ICU patients [5–7]. However, other studies did not find a significant impact of the pandemic on CLABSI rates [8], suggesting that the enhanced infection prevention controls implemented in response to COVID-19 were associated with local reductions in HAIs. Adding to this, few studies have examined the rate of CLABSI among COVID-19 positive and negative patients during the same period of hospitalization. The objectives of the current study were to assess the incidence rates of CLABSI among patients admitted to different types of ICU before, and during, the pandemic, and to compare the CLABSI trends between patients with and without a diagnosis of COVID-19.

## Methods

### Study design

A retrospective surveillance study was carried out in two phases between January 2018 and December 2021, before the start of the pandemic (pre-pandemic period January 2018–December 2019), and during the pandemic period January 2020–December 2021). The study was approved by the IRB committee at King Abdullah International Medical Research Center (KAIMRC), protocol number SP21R-236-05.

### Setting

The study was conducted at different ICUs of King Abdulaziz Medical City (KAMC), which is a tertiary care centre located in Riyadh, Saudi Arabia with approximately 1 100 beds of which 185 are dedicated to intensive care [9] including adult, paediatric, and neonatal units. KAMC provides healthcare services for about 1 000 000 Saudi National Guard soldiers, employees and their families.

### Population

All patients admitted to the ICU with a central line and diagnosed as CLABSI according to NHSN criteria were included. ICU patients without a central line and those admitted to wards were excluded.

### Sample size and sampling

It was estimated that at least 15 000 central line days of follow-up were required to detect a CLABSI rate of 2.70 per 1 000 line days during COVID-19 with a 95% confidence interval (CI) of 1.35 per 1 000 central line days.

### Surveillance methodology

The surveillance methodology of NHSN [10] and the Gulf Cooperation Council Center for Infection Control [11] were used. CLABSI was defined as patients with a positive blood culture, with or without symptoms who had a central line for two or more days. Central lines were either inserted at the ICU of stay at other locations, such as interventional radiology and to a lesser extent, in the emergency department, or surgical rooms. Lines were inserted by physicians of insertion units, with no special vascular access team. The diagnosis of CLABSI events was made by Infection Control staff, who also monitored adherence of practice with insertion and maintenance of lines, as per the standards of NHSN [10]. Three laboratory-confirmed bloodstream infection (LCBI) criteria were recognized as per standard definitions [10, 11].

### Outcomes

The outcomes studied were the CLABSI rates per 1 000 central line days, central line utilization, CLABSI case fatality, and average ICU and hospital stay during the study period. For the pandemic phase, we identified patients with confirmed COVID-19 infection from the study population based on a positive SARS-CoV-2 Reverse transcription polymerase chain reaction (RT-PCR) on a nasopharyngeal swab as guided by the Saudi Centers for Disease Control and Prevention guidelines.

### Data collection

Data were extracted from the surveillance data collected by the Infection Prevention and Control (IPC) department at KAMC, in addition to electronic medical files using a standardized form. The collected data comprised demographic information, type and location of ICU, admission comorbidities and ventilator use, length of stay in ICU, and central line information. The latter included data on the number and type of central lines, their site of insertion and lumen of the line. Additionally, data on blood stream infection (BSI), CLABSI diagnostic types, and microbiological test results.

### Data analysis

Categorical variables were presented as frequencies and percentages, and continuous variables as means and standard deviations (SD). CLABSI rates were expressed per 1 000 central line days. The clinical characteristics of the patients with CLABSI between the pre- and pandemic periods were compared using Chi-square or Fisher exact test as appropriate while CLABSI rates were compared between the two periods using the *Z*-test for event-time data. A *p*-value <0.05 was considered as significant and all *p*-values were two-tailed. SPSS (Version 25.0., IBM Corp., Armonk, NY) was used for all statistical analyses.

## Results

In total, 156 CLABSI events and 46 406 central line days were recorded; 52 and 22 447 (respectively) in the pre-pandemic period, and 104 and 23 959 (respectively) during the pandemic. The average age of all patients was 50 years (SD 28.5) with 51% females. The great majority (81%) of all patients were from adult ICUs, and mainly from the medical-surgical ICU (26%) (Table 1). One-third of the total study population had confirmed COVID-19 infection, and 50% had one or two comorbidities. The rate of death during hospitalization was 59.6%; and 69.1% of these had a clinically significant BSI. Patients in the pandemic period were older (mean age 57 ± 25.1 vs. 36.9 ± 30.2 in the pre-pandemic), had more use of mechanical ventilation (82.5% vs. 53.2%), and were diabetic (50% vs. 25%).

**Table 1.** Demographic and clinical characteristics of the patients with CLABSI before and during COVID-19 pandemic

	Pre-pandemic	Pandemic	Total	*p*-value
Age	36.9 ± 30.2	57.2 ± 25.1	50.4 ± 28.5	<0.001
Gender				
Male	24 (46.2%)	51 (49.0%)	75 (48.1%)	0.734
Female	28 (53.8%)	53 (51.0%)	81 (51.9%)	
Facility				
KASCH	23 (44.2%)	33 (31.7%)	56 (35.9%)	0.125
NGHA–Riyadh	29 (55.8%)	71 (68.3%)	100 (64.1%)	
Location				
Adult ICU	35 (67.3%)	92 (88.5%)	127 (81.4%)	0.001
Paediatric ICU	16 (30.8%)	8 (7.7%)	24 (15.4%)	
Neonatal ICU	1 (1.9%)	4 (3.8%)	5 (3.2%)	
Unit				
Adult medical–surgical ICU	15 (28.8%)	27 (26.0%)	42 (26.9%)	0.003
Cardiovascular ICU–Paediatric	8 (15.4%)	2 (1.9%)	10 (6.4%)	
General ICU	4 (7.7%)	8 (7.7%)	12 (7.7%)	
Medical ICU	6 (11.5%)	23 (22.1%)	29 (18.6%)	
Neonatal ICU	1 (1.9%)	4 (3.8%)	5 (3.2%)	
Paediatric ICU	8 (15.4%)	6 (5.8%)	14 (9.0%)	
Respiratory ICU	0 (0.0%)	12 (11.5%)	12 (7.7%)	
Trauma ICU	5 (9.6%)	9 (8.7%)	14 (9.0%)	
Others	5 (9.6%)	13 (12.5%)	18 (11.5%)	
Hospitalization death				
No	23 (44.2%)	40 (38.5%)	63 (40.4%)	0.489
Yes	29 (55.8%)	64 (61.5%)	93 (59.6%)	
BSI contributed to death				
No	6 (37.5%)	15 (28.8%)	21 (30.9%)	0.546
Yes	10 (62.5%)	37 (71.2%)	47 (69.1%)	
COVID infection				
No	47 (100.0%)	49 (50.5%)	96 (66.7%)	<0.001
Yes	0 (0.0%)	48 (49.5%)	48 (33.3%)	
Ventilator use				
No	22 (46.8%)	17 (17.5%)	39 (27.1%)	<0.001
Yes	25 (53.2%)	80 (82.5%)	105 (72.9%)	
Number of comorbidities				
None	7 (13.5%)	21 (20.2%)	28 (17.9%)	0.155
One or two	32 (61.5%)	47 (45.2%)	79 (50.6%)	
Three or more	13 (25.0%)	36 (34.6%)	49 (31.4%)	
Types of comorbidities				
Cardiovascular diseases	34 (65.4%)	64 (61.5%)	98 (62.8%)	0.639
Diabetes mellitus	13 (25.0%)	52 (50.0%)	65 (41.7%)	0.003
Chronic pulmonary diseases	13 (25.0%)	20 (19.2%)	33 (21.2%)	0.406
Malignant diseases	9 (17.3%)	17 (16.3%)	26 (16.7%)	0.879
Chronic kidney disease	9 (17.3%)	18 (17.3%)	27 (17.3%)	>0.99
Chronic liver diseases	10 (19.2%)	8 (7.7%)	18 (11.5%)	0.033

**Figure 1.**
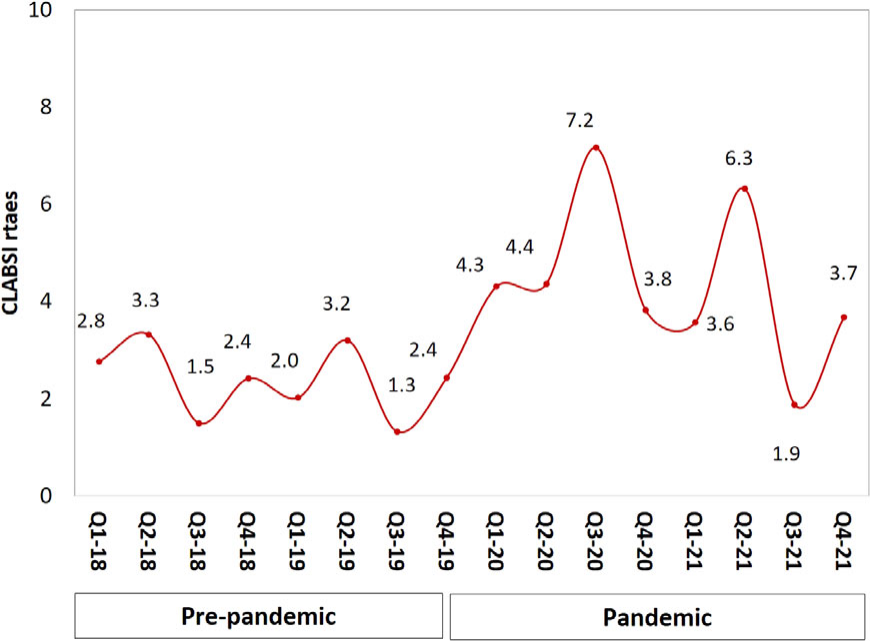
CLABSI rate per 1 000 central line days by quarter and year.

**Figure 2.**
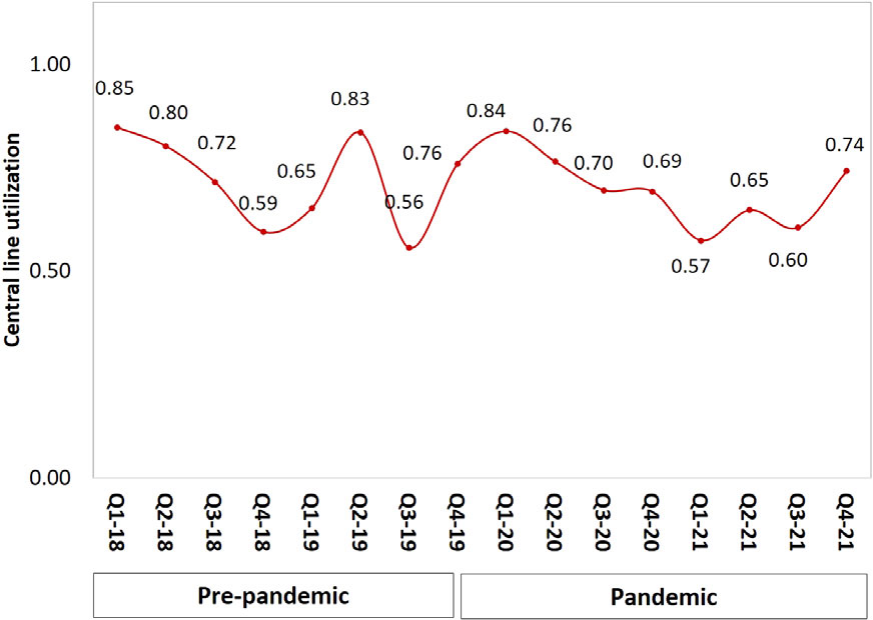
Central line utilization by quarter and year.

**Figure 3.**
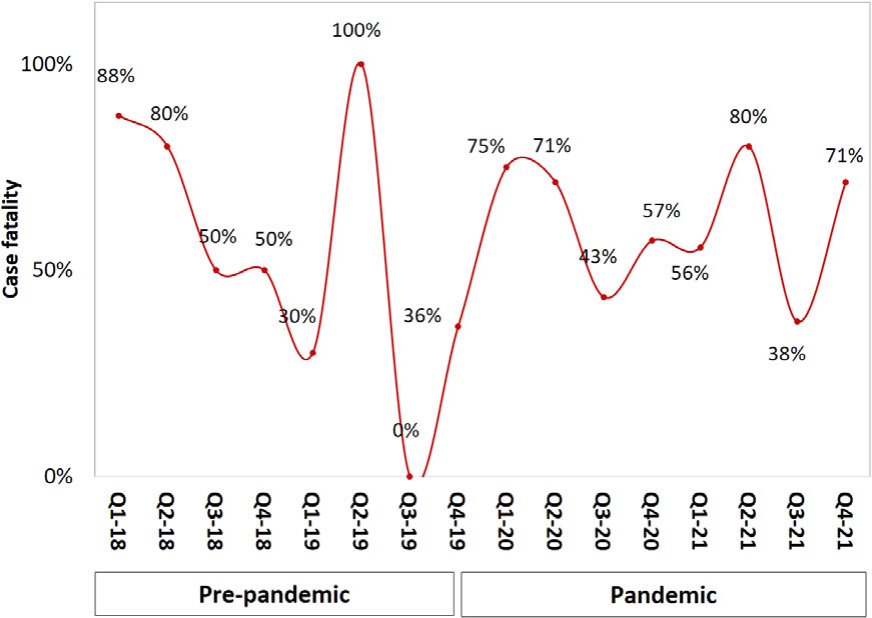
CLABSI case fatality by quarter and year.

**Figure 4.**
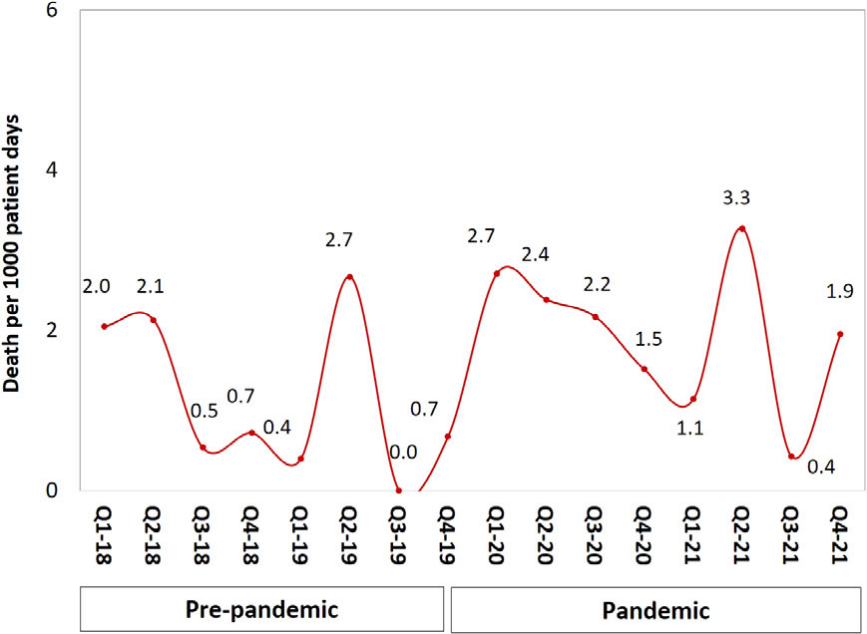
Overall mortality by quarter and year.

CLABSI rates increased by 2.02 per 1 000 central line days during the pandemic (from 2.32 to 4.34, *p* < 0.001; Table 2) but line utilization decreased in the same period compared with pre-pandemic (0.67 vs. 0.72 (*p* < 0.001). Figure 1 illustrates CLABSI rates per 1 000 central line days during both study periods whereby at the end of 2021 the rate was 3.7 per 1 000 central line days, compared with 2.4 at the end of 2019. Likewise, central line utilization also declined during the pandemic period (Figure 2). The CLABSI case fatality was almost zero in the third quarter of the year 2019 but significantly increased during the pandemic period (Figure 3). This was consistent with an increase in overall mortality of 0.86 per 1 000 patient days (from 0.93 to 1.79, *p* = 0.003; Table 2 and Figure 4). The average hospital stay was significantly lower in the pandemic phase (73.5 ± 124.7) than in the pre-pandemic phase (136.4 ± 230.1) (*p* = 0.002).

**Table 2.** CLABSI rates and other related outcomes before and during COVID-19 pandemic

	Pre-pandemic	Pandemic	Total	*p*-value
Events:				
Number of CLABSI events	52	104	156	–
Number of death events	29	64	93	–
Denominators:				
Central line days	22 447	23 959	46 406	–
Patient days	31 253	35 815	67 068	–
Outcomes:				
CLABSI rate per 1 000 central line days	2.32	4.34	3.36	<0.001
Central line utilization	0.72	0.67	0.69	<0.001
CLABSI case fatality	55.8%	61.5%	59.6%	0.489
Mortality per 1 000 patient days	0.93	1.79	1.39	0.003
Average ICU stay (days)	41.9 ± 43.7	47.0 ± 101.9	45.2 ± 86.3	0.471
Average hospital stays (days)	136.4 ± 230.1	73.5 ± 124.7	95.5 ± 171.0	0.002
Average central line days (days)	24.7 ± 25.8	20.4 ± 27.8	21.9 ± 27.1	0.169

Regarding CLABSI-related characteristics before and during COVID-19 pandemic, there was no difference in the type, site, and number of central line insertions between the two periods (Supplementary Table). For the identified pathogens, *Candida* spp. was significantly higher in the pandemic period (26% vs. 12% *p* = 0.043). CLABSI-related outcomes during the pandemic were assessed with the total central line days reaching 20 777. A comparison of CLABSI rates and other related outcomes by COVID-19 status is shown in Table 3. Both CLABSI rates (6.18 vs. 3.7, *p* = 0.006) and overall mortality (2.72 vs. 1.47, *p* = 0.014), were higher among COVID-19 patients during the pandemic compared with non-COVID-19 patients.

**Table 3.** CLABSI rates and other related outcomes by COVID-19 status during the pandemic

	No COVID	COVID	Total	*p*-value
Events:				
Number of CLABSI events	49	48	97	–
Number of death events	29	34	63	–
Denominators:				
Central line days	13 005	7 772	20 777	–
Patient days	19 713	12 507	32 220	–
Outcomes:				
CLABSI rate per 1 000 central line days	3.77	6.18	4.67	0.006
Central line utilization	0.66	0.62	0.64	<0.001
CLABSI case fatality	59.2%	70.8%	64.9%	0.230
Mortality per 1 000 patient days	1.47	2.72	1.96	0.014
Average ICU stay (days)	58.0 ± 129.9	36.2 ± 64.7	47.2 ± 103.0	0.968
Average hospital stay (days)	89.2 ± 159.4	54.5 ± 74.5	71.6 ± 124.6	0.259
Average central line days (days)	21.6 ± 29.9	18.7 ± 26.7	19.9 ± 27.9	0.859

## Discussion

This study assessed the impact of the COVID-19 pandemic on the CLABSI surveillance rates. The key finding was that CLABSI rates increased by 2.02 per 1 000 central line days during the pandemic period. Additionally, both CLABSI and overall mortality rates were higher among COVID-19 positive patients. Our findings are consistent with a national study which examined the impact of COVID-19 on CLABSI among 78 Ministry of Health hospitals in Saudi Arabia [12] and reported an approximately 16% increase of CLABSI in 2020–2021 compared with the 2019 rates. This was also consistent with other international studies whether examined as multihospital systems or single centre studies [4, 13–15].

There has been increased focus on the impact of the COVID-19 pandemic on HAIs. Previous reports had strongly recommended prioritization of surveillance of HAIs with allocated resources accordingly. Ongoing surveillance and monitoring of HAI rates remain of importance to quantify and assess the impact of pandemics on HAIs [16]. The literature on this topic sheds light on several key factors which contribute to such an increase. One factor is the disturbance in infection control practice due to a pandemic in which the availability of adequately trained staff and diversion of resources present a significant challenge to healthcare systems, particularly in acute hospital units, in order to maintain efficient surveillance activities [2, 17]. This may lead to a decline in adherence to strict infection control practices including proper central line insertion and maintenance techniques. Other factors that have been noted by others indicate that prolonged ICU stays for severe COVID-19 cases increase the risk of CLABSI development [4, 18, 19]. Moreover, wider utilization of immunosuppressive agents and antibiotics increase the risk of infection [3, 12].

Regarding the types of central line infection, no significant change in the distribution of the pathogens was observed. For example, Gram-negative bacteria such as Klebsiella and Pseudomonas were more common than Gram-positive bacteria. Similarly, previous studies done in Saudi Arabia showed that Gram-negative bacteria were the major bacteria causing several HAIs including CLABSI [20, 21]. This may be related to poor environmental cleaning and hand hygiene compliance [20, 21]. However, *Candida* spp. in particular, increased by a notable 13.6% in the pandemic period. This finding correlates with a USA study which documented around a 66% increase in these organisms associated with CLABSI. This increase can be attributed to the long-term use of central venous catheters and broad-spectrum antimicrobials [22]. Furthermore, the use of tocilizumab, an immunosuppressive drug, in patients with COVID-19 may increase the risk of candidemia. It is noteworthy that a systematic review found that secondary infections were slightly higher in patients receiving tocilizumab compared with those receiving standard care, but the finding did reach statistical significance [23].

Our study has some limitations; first, the data were collected from a single centre and cannot be directly extrapolated to other healthcare centres around the country. Nevertheless, our findings are consistent with recent national data. Second, the study design limits directly inferring a causal relationship. Third, the major disruption of hospital functions during the pandemic period and the consequent negative impact on the management and maintenance of catheters may have confound the findings. Lastly, as it was based on surveillance data, some specific CLABSI risk factors were unavailable.

In conclusion, the COVID-19 pandemic was associated with a substantial increase in CLABSI-associated morbidity and mortality which was likely due to the clinical complexity of hospitalized patients during the period. Patients with COVID-19 were at higher risk of CLABSI-associated morbidity and mortality. As most CLABSI cases are possibly preventable with proper aseptic techniques, adequate training, and surveillance, maintaining such activities during a pandemic is even more critical to reduce the burden of HAIs.

## Supporting information

Alshamrani et al. supplementary material Alshamrani et al. supplementary material

## Data Availability

The data supporting this study’s findings are available from King Abdul-Aziz Medical City. Data are available from the corresponding author on reasonable request with the permission of King Abdul-Aziz Medical City.

## Supplementary material

For supplementary material accompanying this paper visit http://doi.org/10.1017/S0950268824000736. click here to view supplementary material
