# Supplementary material for: Risk of central line-associated bloodstream infections during COVID-19 pandemic in intensive care patients in a tertiary care centre in Saudi Arabia
Source: Epidemiol Infect. 2024 Jun 3;152:e95. doi: 10.1017/S0950268824000736 (PMC11736446; doi:10.1017/S0950268824000736)
Supplement: Alshamrani et al. supplementary material [file S0950268824000736sup001.docx]

**Supplementary Table: CLABSI related characteristics before and during COVID-19 pandemic**

|  | **Pre-pandemic** | **Pandemic** | **Total** | **p-value** |
| --- | --- | --- | --- | --- |
| **CLABSI diagnostic types** |  |  |  |  |
| LCBI criterion-1 | 48 (92.3%) | 99 (95.2%) | 147 (94.2%) | 0.634 |
| LCBI criterion-2 | 2 (3.8%) | 3 (2.9%) | 5 (3.2%) |  |
| LCBI criterion-3 | 2 (3.8%) | 2 (1.9%) | 4 (2.6%) |  |
| **Number of central lines** |  |  |  |  |
| Single | 30 (57.7%) | 55 (52.9%) | 85 (54.5%) | 0.851 |
| Two | 17 (32.7%) | 38 (36.5%) | 55 (35.3%) |  |
| Three or more | 5 (9.6%) | 11 (10.6%) | 16 (10.3%) |  |
| **Type of central line** |  |  |  |  |
| Temporary | 61 (77.2%) | 109 (65.3%) | 170 (69.1%) | 0.058 |
| Permanent | 18 (22.8%) | 58 (34.7%) | 76 (30.9%) |  |
| **Site of insertion of central line** | |  |  |  |
| Jugular | 38 (48.1%) | 85 (50.9%) | 123 (50.0%) | 0.763 |
| Subclavian | 5 (6.3%) | 8 (4.8%) | 13 (5.3%) |  |
| Femoral | 15 (19.0%) | 25 (15.0%) | 40 (16.3%) |  |
| Umbilical | 0 (0.0%) | 2 (1.2%) | 2 (0.8%) |  |
| Peripheral percutaneous | 21 (26.6%) | 47 (28.1%) | 68 (27.6%) |  |
| **Lumen of central line** |  |  |  |  |
| Single | 9 (11.4%) | 12 (7.3%) | 21 (8.6%) | 0.284 |
| Multiple | 70 (88.6%) | 153 (92.7%) | 223 (91.4%) |  |
| **Pathogens** |  |  |  |  |
| *Candida* spp. | 7 (12.5%) | 29 (26.1%) | 36 (21.6%) | 0.043 |
| *Klebsiella* spp. | 13 (23.2%) | 20 (18.0%) | 33 (19.8%) | 0.426 |
| *Enterococcu*s spp. | 7 (12.5%) | 25 (22.5%) | 32 (19.2%) | 0.120 |
| *Pseudomonas* spp. | 8 (14.3%) | 9 (8.1%) | 17 (10.2%) | 0.213 |
| Others | 21 (37.5%) | 28 (25.2%) | 49 (29.3%) | 0.100 |
| **MDRO** | 56 | 111 | 167 |  |
| No | 25 (61.0%) | 47 (60.3%) | 72 (60.5%) | 0.939 |
| Yes | 16 (39.0%) | 31 (39.7%) | 47 (39.5%) |  |
| **MDRO** |  |  |  |  |
| MDR *Klebsiella* | 3 (23.1%) | 5 (20.0%) | 8 (21.1%) |  |
| MDR *Pseudomonas* | 4 (30.8%) | 3 (12.0%) | 7 (18.4%) |  |
| CRE | 2 (15.4%) | 4 (16.0%) | 6 (15.8%) |  |
| MDR Acinetobacter | 2 (15.4%) | 1 (4.0%) | 3 (7.9%) |  |
| MDR ESBL | 0 (0.0%) | 2 (8.0%) | 2 (5.3%) |  |
| MRSA | 1 (7.7%) | 0 (0.0%) | 1 (2.6%) |  |
| VRE | 1 (7.7%) | 10 (40.0%) | 11 (28.9%) | 0.167 |

*CLABSI*, Central Line-Associated Bloodstream Infection; *COVID-19*, Coronavirus Disease 2019; *LCBI*, Laboratory Confirmed Bloodstream Infection; *LCBI criterion-1* involved detecting recognized pathogen in one or more blood cultures; *LCBI criterion-2* involved detecting common skin contaminant in two or more blood cultures with all age symptoms (fever, chills, or hypotension); *LCBI criterion-3* involved detecting common skin contaminant in two or more blood cultures with infant symptoms (fever, hypothermia, apnea, or bradycardia); *MDRO*; multidrug resistant organism; *MDR*, multidrug resistant; *CRE*, carbapenem resistant *Enterobacteriaceae*; *ESBL*, extended-spectrum beta-lactamases; *MRSA*, methicillin-resistant Staphylococcus aureus; *VRE*, vancomycin-resistant *Enterococcus*.
